# The Emerging Role of Mechanobiology in Connecting Metabolic and Cardiovascular Diseases: From Fundamentals to Future Therapies

**DOI:** 10.3390/biomedicines14030525

**Published:** 2026-02-26

**Authors:** Agnieszka Kowalik, Patrycja Paszenda, Julia Rydzek, Małgorzata Stanios, Julia Soczyńska, Piotr Gajewski

**Affiliations:** 1 Faculty of Medicine, Wroclaw Medical University, 50-367 Wroclaw, Poland; 2 Student Scientific Organisation, Institute of Heart Diseases, Wroclaw Medical University, 50-367 Wroclaw, Poland; 3 Institute of Heart Diseases, University Clinical Hospital, Wroclaw Medical University, 50-367 Wroclaw, Poland

**Keywords:** mechanobiology, mechanotransduction, shear stress, endothelial dysfunction, vascular remodelling, elastography, biomarkers, cardiovascular diseases, metabolic diseases

## Abstract

Mechanobiology has emerged as a unifying framework for understanding how mechanical forces and tissue physical properties regulate cellular function, metabolism, and disease progression. Mechanical forces are fundamental regulators of cellular behaviour and tissue homeostasis. Growing evidence indicates that disturbances in mechanobiological signalling contribute to both metabolic disorders and cardiovascular diseases, two highly prevalent and interrelated groups of conditions. This review aims to synthesize current evidence on mechanobiological mechanisms linking metabolic dysfunction and cardiovascular pathology, with particular emphasis on shared pathways involved in tissue remodelling, inflammation, and disease progression. Shared pathogenic mechanisms, including chronic low-grade inflammation, oxidative and endoplasmic reticulum stress, and lipotoxicity, further reinforce the bidirectional relationship between metabolic and cardiovascular disorders. Moreover, advances in mechanobiological imaging and the usage of mechanobiological biomarkers are more commonly regarded as promising tools for early detection of the disease and risk stratification. It is worth mentioning that targeting mechanosensitive pathways may support the development of personalised diagnostic strategies and novel therapeutic approaches addressing both metabolic and cardiovascular components of disease, which may result in a breakthrough.

## 1. Introduction

Mechanobiology can be considered an expanding field that is oriented toward investigating how mechanical forces and properties influence biological systems at the cellular, molecular, and tissue levels [1]. It is deeply intertwined with human physiology and development, shaping numerous elements of biological functions, behavioural patterns, and adaptive responses [2]. In recent decades, it has been revealed that the mechanical properties of cells play a significant role in signal transduction, tissue morphogenesis, cell migration, and their fate [3]. Cells were found to be able to generate endogenous mechanical forces—such as traction stresses—induced by cytoskeletal dynamics, which, perceived through mechanotransduction, initiate mechanosensitive feedback loops that are crucial for cellular function, tissue development, and the constant maintenance of homeostasis [1,4]. Interest in mechanobiology has grown alongside the identification of genes and proteins that shape cells and tissues, thereby enabling them to generate and respond to mechanical forces [5]. The signalling pathways activated by mechanical forces are crucial for understanding how physical stimuli generate various biological effects, including altered stem cell activity, differentiation, and functional disuse [2].

Based on those findings, the new perspective on the progression of some diseases, especially cardiovascular (CV) and metabolic diseases, has been taken into consideration and received further examination [1,3]. This direction of research appears to be highly vital because of the fact that metabolic and cardiovascular diseases (CVDs) pose a significant public health challenge across populations worldwide, and conditions related to those disorders contribute heavily to the global healthcare burden [6,7,8]. According to the research conducted by the GBD 2023 CVD Collaborators, it has been stated that the global prevalence of CVDs has increased substantially—the number of cases doubled from 311 million in 1990 to 626 million in 2023, and CVD deaths rose from 13.1 million in 1990 to 19.2 million in 2023 [7]. Moreover, based on a study by Zhang H et al. between 1990 and 2021, the global occurrence of the five major metabolic diseases increased noticeably, rising from 1.6- to 3-fold, with a distinct predominance of Type 2 Diabetes Mellitus (T2DM) and obesity [8].

It is worth highlighting that particular attention should be drawn to this topic, as it may enable the development of more precise and targeted therapies, oriented towards highly personalised approaches. This review explores key areas in which mechanobiology interacts with and shapes the course of CV and metabolic diseases.

A review of the contemporary literature was conducted, focusing on experimental, translational, and clinical studies addressing mechanotransduction in metabolic and CV diseases. Key areas of interest included extracellular matrix (ECM) remodelling, tissue stiffness, cytoskeletal dynamics, mechanosensitive transcription factors, and ion channels. Evidence from imaging studies, biomarker research, and emerging therapeutic approaches targeting mechanobiological pathways was also integrated.

## 2. Mechanobiology in Metabolic and Cardiovascular Diseases

### 2.1. Background

Mechanobiology has become a crucial concept regarding CVDs’ pathogenesis, mainly on the grounds that within the CV system, cells are continuously subjected to mechanical stimuli (e.g., fluid shear stress (FSS) and pressure), greatly shaping the entire cellular function and adaptation [9,10]. Through mechanotransduction mechanisms, biomechanical signals play a vital role in endothelial homeostasis, vascular cells’ phenotypic modulation, and cardiac growth responses [10]. In some cases, when mechanobiological signalling is disturbed or altered, it promotes pathological outcomes, such as atherosclerosis, vascular stiffening, and cardiac remodelling [11,12]. Aortic diseases (e.g., aortic aneurysm, aortic dissection), peripheral artery disease (PAD), valvular heart diseases, coronary artery disease (CAD), and its acute manifestations—acute coronary syndromes (ACSs)—are among the most mechanobiologically driven CV conditions [13].

Aortic diseases, especially thoracic aortic aneurysms and dissections, constitute a major cause of morbidity and mortality across all age groups, affecting both younger and older populations [14]. They are characterised by progressive structural weakening of the aortic wall driven by ECM degradation, elastin fragmentation, and smooth muscle cell dysfunction [13].

PAD is a result of systemic atherosclerosis and is stated to affect over 200 million individuals worldwide [15]. Disturbed shear stress at arterial bifurcations promotes endothelial dysfunction and triggers inflammatory activation, which results in accelerated plaque formation in the lower extremities of the arteries [16].

ACSs are clinically represented by the culmination of complex pathological processes within the coronary arteries. While the spectrum of ACS is clinically defined by a sudden critical reduction in myocardial blood supply—ranging from unstable angina to ST-segment elevation myocardial infarction (STEMI) and non-STEMI (NSTEMI) [17]—the underlying mechanisms are more heterogeneous than traditionally appreciated. These pathogenic drivers include not only the well-described plaque rupture but also plaque erosion and the presence of calcified nodules. Despite significant preventive measures and technological advancements in management, CAD and its acute manifestations continue to impose a substantial burden of morbidity and mortality worldwide [18].

Valvular heart diseases (e.g., calcific aortic valve disease) are characterised by structural remodelling of the valves’ leaflets, which includes fibrosis, calcification, and ECM disorganisation. The changes are progressive and therefore lead to stenosis or regurgitation and, consequently, impaired cardiac haemodynamics. It is worth mentioning that valvular diseases may arise, at least in part, from abnormal expression of mechanosensitive ion channels, which disrupt the normal transduction of mechanical signals essential for valve development and function [19,20].

The term “metabolic diseases” comprises conditions in which metabolic processes are altered abnormally [21]. Insulin resistance (IR), T2DM, obesity, and metabolic-associated fatty liver disease (MAFLD) are disorders most commonly linked to dysfunctional metabolism, which share common risk factors and often occur in tandem [21,22]. As metabolic diseases’ prevalence has increased drastically over the past two decades [22,23], much research has been conducted to better understand their pathophysiology [24]. Among other mechanisms, mechanobiological responses have been regarded as one of the crucial factors shaping cells’ metabolism [24,25].

Diabetes mellitus is characterised by hyperglycaemia. While type 1 diabetes is associated with loss of pancreatic β-cells due to autoimmune attack, T2D is linked to β-cell dysfunction and/or IR [26]. The latter is a condition defined by decreased insulin sensitivity, impaired suppression of hepatic glucose production, and peripheral glucose uptake, frequently leading to hyperinsulinaemia to maintain blood glycaemia [21]. Skeletal muscle is an essential tissue for maintaining glucose homeostasis and, therefore, contributes to systemic IR progression if impaired metabolically [27].

Obesity is yet another disease whose pathogenesis can be linked to mechanobiological dysfunction [28]. Adipocytes, previously regarded as inert cells functioning as a mere energy reservoir, are now identified as an active element in metabolic regulation through diverse mechanisms [28,29]. Hypertrophic and hyperplastic adipocyte growth is widely observed in obese patients [29].

MAFLD is defined by the presence of liver steatosis in at least 5% of hepatocytes simultaneous to metabolic dysfunction such as IR or obesity [30,31]. Hepatic liver metabolism is modulated by fatty acid (FA) export and uptake, β-oxidation, and de novo lipogenesis. If the balance between these pathways is disturbed, hepatic lipid accumulation develops and chronic inflammation starts to form, enhancing fibrotic pathways’ activity [31]. If undetected or not treated properly, MAFLD can lead to metabolic dysfunction-associated steatohepatitis (MASH) [30].

### 2.2. Mechanobiology of Cardiometabolic Interactions—A Conceptual Overview

The intricate and bidirectional relationship between CVDs and metabolic disorders, such as T2D and metabolic dysfunction-associated steatotic liver disease, is underpinned by a complex network of shared pathophysiological mechanisms that extend beyond traditional risk factors like hypertension and dyslipidaemia [32]. This convergence is increasingly understood through the lens of mechanobiology, which examines how mechanical forces and the physical properties of tissues and cells influence development, physiology, and disease [33].

### 2.3. Cell–Cell and Cell–ECM Adhesions

Mechanical and biochemical stimuli have been proven to alter pancreatic tissue function [34]. β-Cells forming pancreatic islets are surrounded by ECM, primarily composed of laminin and networked type IV collagen, with hyaluronan present both within and around the islets [26,34]. The peri-islet ECM can be variable in composition and, thus, mechanical properties, demonstrating a significant impact on β-cells’ survival and insulin secretion [34].

Tissue stiffness is directly reliant on ECM and determined by the collagen type and the level of its cross-linking [26]. In states of metabolic stress, the balance between ECM synthesis and degradation is disrupted, leading to excessive deposition of fibrillar collagens and cross-linking by enzymes such as lysyl oxidase (LOX) [35]. This process increases tissue stiffness, a hallmark of both metabolic disease and CVDs [31,36].

Pancreatic tissue is essentially soft, but in T2D’s pathogenesis it undergoes structural changes due to elevated hyaluronan or collagen type I and III deposition, becoming stiffer and promoting islet dysfunction [34].

Increased tissue stiffness is perceived by cellular mechanosensors, such as integrin adhesion complexes (IACs). These protein networks, forming a link between the ECM and intracellular environment, are composed of plasma membrane adhesion receptors, actin regulators, and adaptor and signalling molecules [26]. Key elements of the transduction complex, however, include integrins binding ECM molecules extracellularly and the cytoskeleton intracellularly [26,37,38,39]. Transduction by the IACs’ signal then alters cytoskeletal formation, leading to changes in the spatial organisation of organelles, enzymes, and cargoes [37]. One of the many integrin-mediated mechanosensing pathways is the one acting on the Rho-associated protein kinase (ROCK). Its activation upregulates the transcription factor β-catenin and regulates insulin gene expression [26,34].

At the tissue level, ECM is the main determinant of skeletal muscle stiffness, which increases in IR. Inflammatory factors, such as transforming growth factor β (TGF-β) and interleukin 1β (IL-1β), induce amplified collagen deposition and fibrosis in insulin-resistant muscles. If IR, muscle cells themselves exhibit increased stiffness through cytoskeletal shifts, with an example being elevated branched F-actin amounts. This cortical actin stiffening may act as a physical barrier for Glucose Transporter Type 4 (GLUT4) storage vesicles (GSVs), disabling its translocation to plasma membranes and, therefore, escalating IR. Moreover, GSV transport may be impaired by IR-induced microtubular polymerisation and stabilisation [27].

Focal adhesions are of crucial importance in detecting biomechanical stimuli. If alerted of increased tissue stiffness, they initiate downstream cellular responses involving cytoplasmic signalling pathways and cytoskeleton reorganisation [27]. Mechanical forces transferred through the integrin–cytoskeleton connection reach the mitochondria, altering vimentin and desmin function, thereby regulating mitochondrial performance [27,40,41]. These alterations in mitochondrial dynamics significantly affect insulin responsiveness [27,42].

Mechanical stretching can be mediated through focal adhesions [27]. It has been discovered to impact insulin signalling in adipocytes through GLUT4 translocation, resulting from phosphorylation of Protein Kinase B (AKT), an essential insulin signalling effector. This mechanism can be explained by AKT upregulation attributed to cytoskeletal action. The role of actin filament polymerisation in AKT activity has been widely recognised and proven to be influenced by cyclic stretching. Potential mechanosensors of this process are adverse in action: focal adhesion kinase (FAK) and ROCK [29].

FAK is a kinase that controls focal adhesion/cytoskeleton signalling. Cyclic stretching inhibits its activity, leading to AKT phosphorylation and upregulation. ROCK, on the other hand, terminates AKT activation via modulated actin filament formation [29].

In hepatocytes, matrix stiffness can directly activate FAK and the downstream RhoA/ROCK pathway [38,43]. There are two ROCK isoforms (ROCK1 and ROCK2), which are regulated differently: ROCK1 promotes traction force, while ROCK2 regulates cytoskeletal dynamics [38,44]. RhoA/ROCK signalling has been proven essential for Yes-associated protein (YAP)/transcriptional coactivator with PDZ-binding motif (TAZ) activation [38].

Similar to metabolically active tissues, CV structures are profoundly dependent on ECM composition and integrin-mediated force transmission [45].

In large vessels, progressive collagen I/III accumulation, elastin fragmentation, and increased cross-linking elevate wall stiffness, fundamentally altering vascular smooth muscle cells’ mechanosensing [46]. Aortic diseases (e.g., aortic aneurysm and aortic dissection) result from complex interactions between ECM remodelling, vascular smooth muscle (VSCM) dysfunction, and abnormal biomechanical forces. Chronic exposure to elevated wall stress, hypertension, and disturbed flow patterns disrupts endothelial mechanosensing and impairs ECM–VSMC signalling, leading to elastin fragmentation, collagen reorganisation, increased matrix metalloproteinase activity, and progressive medial degeneration. VSMCs exhibit marked phenotypic plasticity, transitioning from a contractile to a synthetic state in response to mechanical, inflammatory, or genetic stimuli, which further compromises the structural integrity of the aortic wall. Dysregulation of key molecular pathways—including TGF-β/SMAD, Hippo/YAP, and Notch, along with mutations in genes encoding contractile proteins (e.g., ACTA2, MYH11, MYLK, PRKG1) or ECM components (e.g., FBN1, COL3A1, LOX)—contributes to wall weakening and susceptibility to dissection [47]. Moreover, inflammatory signalling, particularly mediated by TNF-α, activates the metalloproteinases ADAM10 and ADAM17, which cleave the extracellular domain of VE-cadherin (VEC). VEC is a key endothelial adhesion molecule responsible for maintaining vascular barrier integrity. This process results in the release of soluble VEC (sVEC) and contributes to endothelial barrier disruption and increased vascular permeability. In vitro experiments in human aortic endothelial cells (ECs) confirmed that TNF-α–induced VEC shedding is ADAM10/17-dependent and directly associated with impaired endothelial monolayer integrity. In patients with aortic aneurysm and Stanford type B aortic dissection, circulating sVEC levels were detectable but not significantly different from those observed in other vascular diseases. However, plasma sVEC concentrations were positively correlated with TNF-α and, more strongly, with ADAM10 levels, particularly in chronic aortic dissection. These findings suggest that VEC proteolysis reflects inflammatory and proteolytic activity relevant to aortic wall pathology [48].

Calcific aortic valve disease (CAVD) is characterised by maladaptive remodelling of the ECM, which actively regulates the behaviour of valvular interstitial cells (VICs) and endothelial cells (VECs) through mechanobiological signalling. In the fibrosa layer, fragmentation of elastin, disorganisation of type I and III collagen, and accumulation of proteoglycans (e.g., biglycan, decorin, and versican) alter the spatial presentation of growth factors and pro-inflammatory cytokines (e.g., TGF-β and TNF-α), thereby amplifying local pro-calcific and pro-fibrotic signals. Mechanical alterations in the ECM, including increased leaflet stiffness (15–25 kPa), engage integrin-mediated adhesion complexes in VICs, triggering cytoskeletal reorganisation and mechanotransduction. These processes activate various signalling pathways (e.g., TGF-β/Smad, Wnt/β-catenin, and MAPK/ERK1/2), promoting VICs’ differentiation into myofibroblast- and osteoblast-like phenotypes, with upregulation of α-SMA, ALP, Runx2, osteocalcin, and osteopontin [49].

Proteoglycans and glycosaminoglycans modulate the bioavailability of TGF-β and FGF, facilitating matricrine signalling, while collagen and elastin fragments act as matricellular cues to influence VIC differentiation and apoptosis [49,50]. ECM-associated proteins such as periostin, tenascin-C, and chondromodulin-I further regulate angiogenesis and matrix remodelling via MMP-2, MMP-9, and cathepsins, with layer-specific effects on osteogenic differentiation: periostin promotes angiogenesis and ECM remodelling, whereas chondromodulin-I inhibits neovascularization, limiting inflammation and mineralization. Supraphysiological haemodynamic forces, including disturbed flow and increased bending stress, enhance TGF-β signalling and osteogenic marker expression in the fibrosa, which may explain the preferential localisation of lesions to regions experiencing the highest mechanical load [49].

### 2.4. Transcription Factors

YAP/TAZ are clearly associated with mechanotransduction [26,27,28,51]. They bind to enhancer elements through the transcriptional enhanced associate domain (TEAD), promoting the progression of the cell cycle and pro-fibrotic programs [26,52]. Normally, their activity is restrained by Hippo signalling via a cascade of protein phosphorylation, which ensures cytoplasmic retention followed by degradation [26].

YAP/TAZ activation has been proven to be dependent on tension in the actomyosin cytoskeleton, Rho GTPase, and FAK activity. In pancreatic tissue, YAP expression varies during different states of organ development, peaking at early stages and promoting cell proliferation [26]. In skeletal muscles, YAP regulates lipotoxicity and FA oxidation, whereas TAZ affects AKT and GLUT4 signalling. YAP has been proven to impact myocardial fibrosis via PIEZO1; skeletal muscle may present similar mechanisms [27]. In adipose tissue, YAP/TAZ promotes differentiation of mesenchymal stem cells through the Hippo pathway [28,51]. YAP and TAZ may also increase adipocyte GLUT1 expression, thereby stimulating glucose metabolism [51].

**Figure 1:**
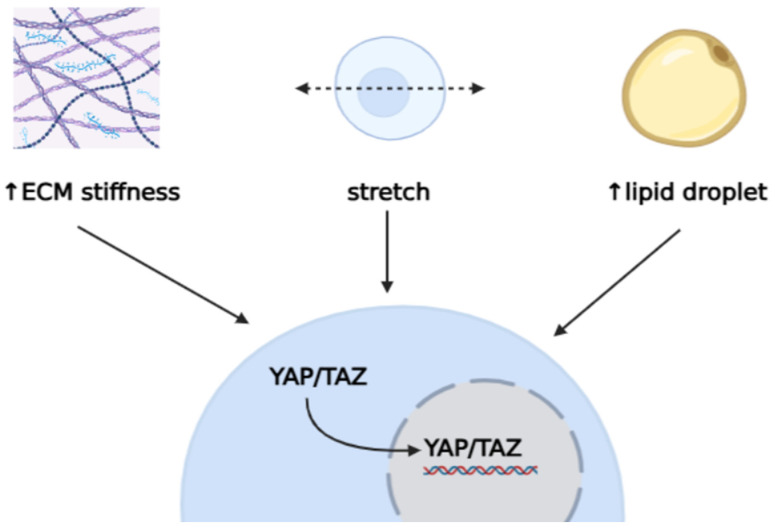
Exemplary mechanisms leading to YAP/TAZ nuclear translocation [28,51]. Created in BioRender. Paszenda, P. (2026) https://BioRender.com/3zu1arm.

YAP/TAZ have been proven to respond to adipocyte stretching or spreading, tissue stiffness, and FSS by yet-unrecognised mechanisms (Figure 1) [51]. It has, however, been recognised that these parameters impact YAP/TAZ localisation, essential to its activity [28]. Amid adipogenesis, YAP/TAZ is relocated from the nucleus to the cytoplasm. The cytoplasmic placement of TAZ inhibits β-catenin’s nuclear entry, favouring adipocyte differentiation. It has been reported that, with a lesser cell spread, YAP is phosphorylated and kept inactive in the cytoplasm, thereby promoting adipogenesis regardless of environmental conditions. However, overexpression of YAP in adipose stem cells has been shown to increase adipogenesis and obesity in mice through the suppressed TAZ activity mediated by negative feedback on the Hippo pathway [51]. In hepatocytes, lipid droplet size has been proven to affect cell stiffness and, in turn, impact YAP localisation, as large droplets have been associated with greater nuclear YAP [30,53,54]. Accumulation of small lipid droplets did not alter YAP’s nuclear localisation. This effect has been attributed to the nucleus displacement or deformation by large lipid droplets, which then alter hepatocyte mechanotransduction [54].

In terms of CVDs, CAD’s pathophysiology has been strongly associated with transcription factors that play a vital role in regulating endothelial function, vascular inflammation, and atherogenesis [55]. Genome-wide association studies (GWASs) have identified numerous genetic variants associated with CAD, influencing both coding and non-coding regions and modulating gene expression in vascular tissues [56,57]. GATA-binding protein 2 (GATA2) promotes monocyte adhesion and endothelial activation via VCAM-1 upregulation, facilitating early atherosclerotic lesion formation. Nuclear factor-kappa B (NF-κB) drives inflammatory signalling and VEGF-mediated angiogenesis, contributing to plaque progression and vascular remodelling. Activating transcription factor 3 (ATF3) regulates macrophage activity and ECM remodelling through the PI3K-Akt-MMP pathway, modulating plaque stability. Signal transducer and activator of transcription 3 (STAT3), including its mitochondrial functions, regulates endothelial dysfunction, inflammation, and immune responses in atherosclerotic vessels [55]. Moreover, a potential genetic predisposition has been observed, with polymorphisms in the transcription factor MEF2A gene being more frequent in this group of patients. This may suggest a role in endothelial dysfunction and atherosclerotic progression [58]. Putting everything together, these transcription factors collectively regulate the molecular processes that promote endothelial dysfunction, chronic vascular inflammation, and plaque formation in CAD.

### 2.5. Ion Channels

Tissue stiffness may alter not only via YAP/TAZ, but also through different mechanisms. PIEZO ion channels have become key elements in CV and metabolic mechanobiology since their identification in 2010. They were initially recognised as the ion channels responsible for touch and proprioception in mammals [59,60]. PIEZO1 and PIEZO2 are broadly expressed across numerous mammalian tissues and organs—PIEZO1 is primarily expressed in non-neuronal tissues such as the lungs, bladder, kidneys, and skin, whereas PIEZO2 is mainly localised in sensory cells, including trigeminal and dorsal root ganglion neurons as well as Merkel cells [59,61,62]. PIEZO channels function as prototypical mechanosensitive channels with mechanical forces triggering the opening of a non-selective cationic pore. PIEZO1 channels have emerged as central mechanotransducers with respect to ECs, as they are located at the apical membrane of ECs and interendothelial junctions between them [59]. They respond to diverse forces, such as shear stress, cellular compression, membrane tension, cell swelling, and ultrasound. Moreover, they exhibit instant activation and inactivation kinetics, although this effect may be influenced by cell type and specific membrane mechanics [59,63,64,65].

PIEZO1 activity is upregulated by the fibrosis of the pancreatic peri-islet ECM and reported to promote islet dysfunction [66,67]. PIEZO1 is a non-selective cation channel that is gated mechanically, thereby being responsive to stimuli such as stretching, static pressure, and shear stress [34,66,67]. Thus, its opening may be triggered by the fluctuations in the ECM stiffness, altering Ca^2+^ homeostasis, thereby resulting in insulin secretion dysfunction [34].

The elementary mechanism overseeing insulin secretion remains dependent on serum glucose level changes [68]. The β-cell response to glucose is driven by the closure of the ATP-gated potassium channels (K_ATP_), cell depolarisation, and Ca^2+^ influx [34,69]. However, while K_ATP_ closure is crucial for glucose-stimulated insulin secretion (GSIS), alone it is insufficient to depolarise the cell membrane, as its potential is determined by the overall balance of ionic currents [69].

**Figure 2:**
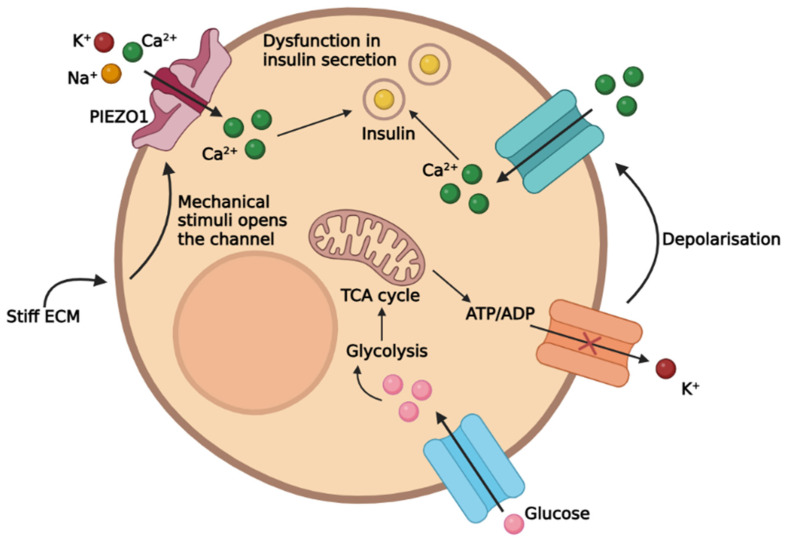
Mechanosensing in pancreatic β-cells [34]. Created in BioRender. Paszenda, P. (2026) https://BioRender.com/i7pq0yk.

Recent studies have shown that K_ATP_-reliant insulin secretion may be regulated by PIEZO1 as it alters calcium homeostasis [34]. Moreover, PIEZO1 closure enhances ATP production and promotes glycolysis via phosphofructokinase (PFK) activation [34,70]. Its Ca^2+^-mediated upregulation is of crucial importance to GSIS, further confirming that increased pancreatic stiffness is involved in dysfunctional insulin secretion (Figure 2) [34].

Ca^2+^ dynamics and ATP hydrolysis drive the interaction of actin and myosin, key to active skeletal muscle contraction. AMP-activated protein kinase (AMPK) and Ca^2+^-reliant signalling pathways are of crucial importance for muscle contraction; moreover, they act as mediators of contraction-induced GLUT4 translocation to the plasma membrane [27,71].

Muscle fibres’ contractility and excitability are sustained due to the external calcium influx [27]. In IR patients, elevated resting and peak Ca^2+^ levels have been observed due to impaired Ca^2+^ handling and PIEZO1 activity [27,72]. Consequently, several pathways and enzymes are activated, further altering glucose metabolism and insulin sensitivity [27].

PIEZO1 is crucial not only for muscle contraction but also for glucose uptake. It affects GSV’s translocation to the plasmatic membrane via F-actin, and its dysfunction is suggested to contribute to skeletal muscle IR [27,72]. Additionally, the PIEZO1/Krüppel-like factor 15 (KLF15)/IL-6 pathway has been recognised to influence muscle metabolism and GLUT4 translocation, offering another mechanism responsible for muscle IR progression [27]. Worth mentioning is the role of exercise in enhancing calcium homeostasis and, therefore, improving muscle function and insulin sensitivity [27,72].

**Figure 3:**
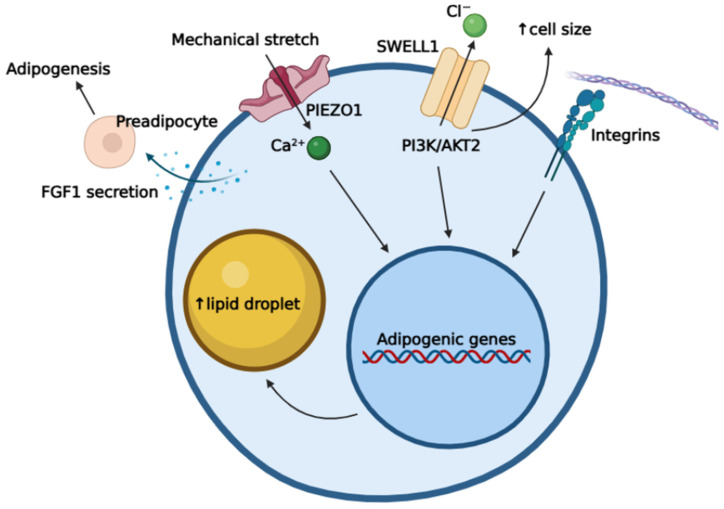
Mechanotransduction in adipocytes [28,51]. Created in BioRender. Paszenda, P. (2026) https://BioRender.com/3c9h3pt.

PIEZO channels can be found also in visceral, perivascular, and subcutaneous adipose tissue [28]. When stimulated, they encourage adipocytes to release adipogenic fibroblast growth factor 1 (FGF1), fostering PIEZO1-dependent adipogenesis (Figure 3) [51,73]. A variety of mechanical stimuli have been proven to activate adipocyte PIEZO1, including cell migration, FSS, osmotic swelling, direct cell membrane indentation or its compression, and forces at cell–matrix or cell–cell junctions [28,73]. Adipocyte hypertrophy, through volumetric expansion, has also been shown to promote PIEZO1 activity [51].

The growing evidence indicates that PIEZO proteins, mainly PIEZO1, are expressed in some of the CV cells: mainly ECs, cardiomyocytes, cardiac fibroblasts, and vascular smooth muscle cells [59,74]. As a result, PIEZO channels are increasingly linked to CV physiology and pathology, especially in sensing and transducing haemodynamic forces within endothelial and vascular cells, maintaining red blood cell homeostasis, regulating platelet aggregation, and modulating arterial blood pressure [59,60].

ECs form a continuous monolayer lining all blood vessels; therefore, their functions are modulated not only by chemical mediators such as hormones, cytokines, and neurotransmitters but also by mechanical forces generated by blood flow [75]. Due to the location of PIEZO1 on ECs, they are exposed to constant shear stress and can also sense membrane tension or stretching. Moreover, in cases of disturbed blood flow, the signalling from those channels triggers vascular remodelling and dysfunction of the endothelium, as observed in atherosclerosis or hypertension [59]. It is worth mentioning that, in adult vasculature, shear stress and cyclic stretching, captured by PIEZO, influence numerous EC functions, including cell shape and alignment, proliferation and apoptosis, vascular tone, antithrombotic activity, reactive oxygen species balance, and gene expression [59,65].

Endothelial PIEZO1 channels play a key role in aligning ECs with blood flow and regulating adherens junction formation and remodelling, by mediating a force-dependent Ca^2+^ influx at cell–cell contacts [59]. Apart from that, PIEZO1 channels are also essential for leukocyte diapedesis and their movement out of the vascular system [76]. Another important role of PIEZO1 channels is regulating nitric oxide production by many different pathways that coordinate to enhance NO synthesis, contributing to vascular relaxation, capillary stability, and protection against endothelial apoptosis [59].

Beyond their unquestionable role in physiology, PIEZO channels are implicated in a variety of CV pathologies, especially under disturbed or high shear stress [59]. In conditions such as pulmonary hypertension, vascular calcification, and atherosclerosis, abnormal PIEZO1 activation contributes to inflammation, endothelial–mesenchymal transition, barrier dysfunction, thrombosis, and plaque formation [77,78]. In PAD, which is strongly associated with disturbed blood flow and low oscillatory wall shear stress, PIEZO1 activation in ECs, vascular smooth muscle cells, and macrophages may trigger pathways that promote structural changes characteristic of PAD, including arterial stiffening and plaque formation in the arteries. Furthermore, pharmacological inhibition of PIEZO1 has demonstrated anti-inflammatory and anti-fibrotic effects, indicating that modulation of this channel could represent a potential therapeutic strategy in PAD [79]. PIEZO1 dysregulation also impairs endothelial autophagy through YAP activation, further promoting atherogenic phenotypes [80].

Another point worth mentioning is that PIEZO1 is known for contributing to myocardial stiffening and fibrosis through promoting fibroblast-to-myofibroblast conversion and IL-6 production and secretion. Moreover, it activates hypertrophic signalling pathways in cardiomyocytes via mediating Ca^2+^ influx [81].

In the context of a thoracic aortic dissection (TAD), activation of endothelial PIEZO1 channels by triggering Trib-1-mediated degradation of the tight junction protein ZO-1 has been shown to promote the disease. Therefore, inhibiting PIEZO1 stabilises endothelial junctions, which can have a potential therapeutic outcome for TAD [82].

PIEZO1 channels are also known as essential regulators of atrioventricular (AV) valve development. These channels are highly expressed in the AV canal and mediate the transduction of mechanical forces from blood flow into intracellular signals that guide valve morphogenesis. Mutations in PIEZO1 channels result in valve elongation defects and abnormal retrograde blood flow, indicating their critical role in maintaining proper valve structure and function. These findings highlight the crucial role of PIEZO channels as mechanosensors, converting haemodynamic forces into intracellular signals that are vital for the development and proper function of cardiac valves [20].

Taking everything into consideration in the context of PIEZO channels, it can be stated that they are key mechanotransducers in both physiological and pathological vascular and metabolic responses. Therefore, further studies are needed with regard to their vital role in mechanobiology [59,60].

SWELL1, a volume-regulated anion channel, has been identified as another component of the adipocyte mechanosensing apparatus (Figure 3). Its role in tumour progression and immune cell development has been widely recognised; its metabolic regulatory function, however, is a focus of recent studies. Emerging research has established SWELL1 as a protein moderating glucose and lipid metabolism, cell proliferation, apoptosis, and insulin secretion [83].

SWELL1 dysfunction has been shown to disrupt insulin secretion and signalling; moreover, its potential in altering hepatic lipid metabolism has been discovered. Upregulated SWELL1 activity in white adipose tissue has been reported in obese mice fed a high-fat diet, suggesting its involvement in the progression of adipocyte hypertrophy [83].

Another type of mechanosensitive ion channel is transient receptor potential (TRP) channels. Even though the mechanisms underlying TRPs’ response to mechanical stimuli remain not understood thoroughly, several hypotheses have been proposed. Of major importance remain tensional forces generated by ECM and shear stress, both responsible for the channel opening either directly or via actin cytoskeleton or G protein-coupled receptors [26].

Many types of TRPs are widely present in receptor cells [26]. TRPs have been also observed in human pancreatic β-cells; specifically, transient receptor potential melastatin 2 (TRPM2) is a non-selective cation channel enabling Na^+^, K^+^, and Ca^2+^ flow [26,69]. As stated previously, basal calcium concentration is crucial for modulating GSIS [26].

To conclude, metabolic and CV diseases are often linked to altered mechanosensing in cells via various mechanisms. ECM stiffening, along with cytoskeletal and ion channel changes, can be associated with dysregulated mechanisms, further promoting the development of pathological processes.

### 2.6. Shear Stress and Endothelial Function

**Figure 4:**
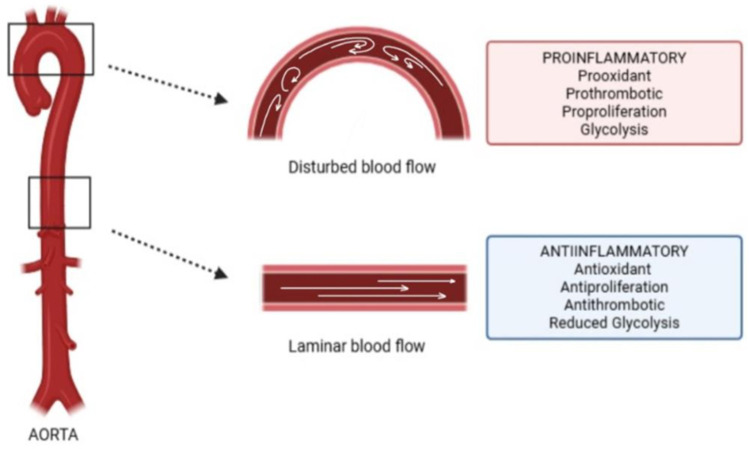
Differences between laminar and disturbed blood flow and their outcomes. Created in BioRender. Paszenda, P. (2026) https://BioRender.com/r3aky48.

The vascular endothelium lines the interior of blood vessels and plays a significant role in preserving vascular integrity and homeostasis while remaining in direct contact with circulating blood [11]. ECs are responsible for creating a selective barrier that controls fluid and biomolecule exchange across the vascular wall. However, when endothelial dysfunction is present, it considerably contributes to severe vascular disorders—for instance, atherosclerosis or thrombosis [11,84]. ECs are highly sensitive to FSS, which is represented by frictional forces generated by the flow of blood at the surface of a vessel wall. Mechanobiology encompasses the processes by which ECs are able to detect, transmit, and most importantly respond to mechanical forces, which is beneficial in the regulation of cellular behaviour [11]. Arterial geometry, in combination with the pulsatile nature of the blood flow, shapes the local haemodynamic patterns [11,85]. Straight arterial segments are typically exposed to a stable, laminar blood flow that is dependent on the cardiac cycle, whereas the curved segments of the vessels are more likely to experience disturbed flow patterns (Figure 4) [11,86]. ECs, which are exposed to laminar shear stress, were found to adopt anti-inflammatory, antioxidant, and anti-proliferative phenotypes, combined with a supressed endothelial-to-mesenchymal transition (EndMT) and, moreover, reduced glycolytic activity, lipid infiltration, and diminished leukocyte adhesion and transmigration [11,87]. Laminar shear stress sustains vascular homeostasis by promoting the expression of vasodilatory mediators—for instance, endothelial nitric oxide synthase (eNOS) and nitric oxide (NO). At the same time, it contributes to the downregulation of pro-atherogenic genes, which encode adhesion molecules and chemokines such as vascular cell adhesion molecule-1 (VCAM-1), intracellular cell adhesion molecule-1 (ICAM-1), and monocyte chemotactic protein-1 (MCP-1), responsible for the adherence of circulating blood elements [11,85,88]. In comparison, the ECs exposed to disturbed blood flow were shown to have pro-inflammatory, pro-oxidant, and pro-proliferative responses, combined additionally with an enhanced EndMT phenotype and glycolysis [11,84]. The molecular mechanisms underlying flow-induced endothelial dysfunction mainly involve the activation of NF-κB, YAP/TAZ, and hypoxia-inducible factor 1α (HIF-1α) pathways [89,90,91,92].

In the context of Abdominal Aortic Aneurysm (AAA), ECs contribute to its pathogenesis via increased oxidative stress, partially resulting from reduced NO bioavailability due to endothelial dysfunction and NADPH oxidase overexpression. They also express adhesion molecules, selectins, and endothelin-1, modulating inflammatory infiltration and oxidative signalling. Inflammatory cells, including monocytes, neutrophils, and lymphocytes, intensify aortic wall degradation by secreting proteolytic enzymes and pro-inflammatory cytokines [93].

The endothelium plays a central role in the pathogenesis of ACS, responding dynamically to changes in wall shear stress (WSS). Local variations in WSS modulate endothelial function through the regulation of genes involved in oxidative stress, inflammation, cell adhesion, and ECM remodelling. Unstable plaques, particularly those with ruptured fibrous caps (RFCs), are exposed to high and heterogeneous WSS, which correlates with the activation of genes such as ADAMTS13, MMP9, and NOS3, driving matrix degradation, thrombus formation, and antioxidant responses. In plaques with intact fibrous caps (IFCs), higher expression of EDN1, TNFα, and LGALS8 is observed under lower WSS, highlighting the dependence of endothelial responses on shear stress magnitude. High WSS acts both directly as a mechanical force promoting plaque rupture and indirectly by altering plaque composition and vulnerability [94].

Based on a study by Fukuyama et al., published in 2023, among 100 ACS patients with OCT-confirmed plaque rupture, regions of rupture exhibited higher WSS than non-ruptured areas. Upstream WSS was linked to upstream ruptures, thinner fibrous caps to downstream ruptures, and peak lateral or central WSS corresponded to lateral and central ruptures. These findings indicate that elevated WSS is associated with both the longitudinal and circumferential locations of plaque rupture, suggesting a key role of shear stress in the destabilisation of plaques [95].

### 2.7. Vascular Remodelling

Vascular wall remodelling is crucial in the development and progress of CV disorders (e.g., atherosclerosis, hypertension, and stroke). Remodelling is a complex process involving CV cell migration, hypertrophy, proliferation, apoptosis, and alterations in cellular phenotype, structure, and function [9,96].

A wide range of mechanosensitive receptors are localised in or on the vascular cell membrane (e.g., integrins, ion channels, junctional proteins, growth factor receptors, receptor tyrosine kinases) and remain in direct correlation with cytoskeletal filaments, which form the cytoskeleton [9,97,98,99]. The primary role of the cytoskeleton is to maintain the shape and structure of the cell in order to enable specific cellular functions [100]. It has been proven that shear stress and different blood flow patterns influence the cytoskeletal assembly and behaviour [9]. In addition to structural changes, mechanical stimuli indirectly affect gene transcription, because the cytoskeleton regulates gene transcription through nucleocytoplasmic shutting of mechanosensitive transcriptional activators [101]. This proves that mechanical forces are in a strong correlation with cellular function and structure as a result of their modulation of the cytoskeleton [9].

Moreover, proteins associated with the nuclear envelope (NE) have been found to act as direct sensors of mechanical forces and, subsequently, regulators of gene expression [102]. Therefore, those molecules participate in shear stress-mediated mechanotransduction, which regulates the proliferation, apoptosis, and migration of ECs. However, in comparison with the cytoskeleton, the role of nuclear mechanotransduction is not fully understood and requires further research [9].

Vascular remodelling represents a key pathophysiological feature of CVD and is strongly regulated by mechanical stimuli, because ECs mainly detect these forces and convert them into biochemical signals, which control vascular structure and function [9].

### 2.8. Summary

In conclusion, CV and metabolic disorders are inextricably linked by a dense, reciprocally reinforcing network of biomechanical and biochemical mechanisms. Pathological ECM remodelling and tissue stiffening, chronic low-grade meta-inflammation, oxidative and ER stress, lipotoxicity, and endothelial dysfunction do not operate in isolation but form an integrated pathophysiological axis [103]. This shared mechanistic landscape underscores that the heart, vessels, liver, pancreas, and adipose tissue communicate constantly through biomechanical forces and soluble mediators, creating a self-perpetuating cycle of disease [104]. Understanding these connections through the framework of mechanobiology is essential, as it reveals novel therapeutic targets—such as YAP/TAZ signalling, specific ECM components, or mechanosensitive ion channels—that could simultaneously address the mechanical and metabolic facets of these intertwined epidemics, paving the way for future holistic therapies [66,105].

## 3. Mechanobiological Imaging and Biomarkers

### 3.1. Methods for Assessing Tissue Stiffness

In this section, we focus primarily on imaging and biomechanical assessment methods that are gaining clinical relevance in CV and metabolic diseases. Selected experimental techniques are briefly mentioned to illustrate the links between microscale mechanics and macroscale observations.

Elastography is a modern, non-invasive medical imaging technique that allows the assessment of tissue stiffness and elasticity, complementing traditional ultrasound (US) and magnetic resonance imaging (MRI) [106]. Elastography is based on measuring the response of tissues to applied mechanical force—this can be pressure from an ultrasound transducer, an acoustic wave, or mechanical vibrations generated by a special device [107]. There are two main methods of US elastography: strain imaging, which involves assessing tissue deformation under compression, and shear wave imaging, which analyses the speed of shear wave propagation in tissue (the greater the stiffness, the faster the wave travels) [108]. The results are presented in the form of colour maps (elastograms), which can be qualitative or quantitative, and stiffness values are most often expressed in kilopascals (kPa) [106].

Magnetic resonance elastography (MRE) is based on a similar principle but uses MRI sequences to generate and detect mechanical waves [107]. MRE allows for the assessment of the stiffness of deeper organs, such as the liver or brain, and is particularly valued for its high repeatability and the ability to quantitatively measure the mechanical properties of tissues [109,110]. Both US and MRI elastography are widely used in the diagnosis of diseases of the liver (e.g., assessment of the degree of fibrosis), breast, thyroid, prostate, muscles, and cancerous lesions, as well as in monitoring the effects of treatment [106,107,111].

The advantages of elastography include non-invasiveness, the possibility of repeating the examination, and providing information not available in classical morphological imaging [106,107]. Limitations include operator dependence (especially in compression methods), difficulties in assessing deep tissues (in the case of US), and the influence of technical and anatomical factors on the reliability of measurement. Despite these limitations, elastography is developing dynamically and is increasingly becoming an important element of diagnostic imaging [108].

Elastography enables the assessment of myocardial stiffness and vascular wall stiffness, which is important in the diagnosis and differentiation of cardiomyopathy, heart failure, hypertension, and atherosclerosis [112]. Techniques such as shear wave elastography allow for quantitative measurement of myocardial stiffness, which can support the assessment of diastolic function and the identification of early changes in diseases such as amyloidosis or hypertrophic cardiomyopathy [112,113]. Vascular elastography allows for the assessment of arterial wall biomechanics and the identification of atherosclerotic plaques with an increased risk of rupture, which is prognostically important in the prevention of stroke and heart attack [111,114,115]. Elastography has been shown to be more sensitive than conventional imaging methods in detecting early pathological changes and in differentiating between stable and unstable atherosclerotic plaques [114,115].

In metabolic diseases, especially non-alcoholic fatty liver disease (NAFLD/MAFLD), elastography is considered the gold standard for non-invasive assessment of the degree of liver fibrosis and steatosis [107,116]. It allows for mass, rapid, and repeatable screening of patients at risk, enabling early detection of advanced fibrosis, which is associated with an increased risk of CV complications [116,117,118]. The high prognostic value of elastography in assessing CV risk has been confirmed, among others, in patients with NAFLD, where higher liver stiffness values correlate with a higher risk of CV events [117,118].

Atomic force microscopy (AFM) is an advanced technique for imaging and measuring the mechanical properties of materials at the nanometre level, widely used in the biological sciences, medicine, and materials science [33,119]. In AFM, a sharp probe mounted on a flexible microcantilever moves over the surface of the sample, recording the interaction forces between the tip and the material under investigation. This allows three-dimensional images of topography and maps of mechanical properties such as stiffness, elasticity, adhesion, and friction to be obtained with a resolution down to a single nanometre [120,121]. AFM enables the study of both hard and soft materials, including cells, bacteria, viruses, proteins and nanoparticles, often under conditions similar to physiological ones [122,123].

AFM can be combined with other methods, such as optical microscopy or spectroscopy, enabling simultaneous analysis of the morphological, mechanical, and functional properties of the systems under study [33,120]. Thanks to this, AFM is used in research on mechanobiology, biointerface design, disease diagnosis, and the development of new materials and drug delivery systems [123].

AFM allows for precise measurement of the stiffness, elasticity, and adhesion of heart cells, vessels, and blood morphotic elements such as erythrocytes and platelets [124,125]. Changes in these parameters are characteristic of many CVDs, including heart failure, cardiomyopathy, atherosclerosis, and arrhythmia [126,127,128]. For example, patients with chronic heart failure have been shown to have increased fibrinogen-binding forces to erythrocytes and changes in cell stiffness, which correlate with a higher risk of hospitalisation and CV complications [124]. AFM also allows for the assessment of the degree of fibrosis and remodelling of the myocardium, which is prognostically significant and may support the personalisation of therapy [128,129].

In metabolic diseases such as T2D, AFM allows the detection of subtle morphological and mechanical changes in erythrocytes, such as increased stiffness, adhesion, and aggregation, which are associated with an increased risk of CV complications [129,130]. These changes can be used as early biomarkers of vascular damage and CV risk in patients with diabetes [131]. AFM also enables the analysis of cell and tissue mechanics in the context of fatty liver disease, IR, or chronic inflammation, which can support early diagnosis and monitoring of disease progression [129,130].

Optical coherence elastography (OCE) is a modern, non-invasive imaging technique that is a functional extension of optical coherence tomography (OCT) [132,133]. OCE enables the mapping and quantitative assessment of biomechanical tissue properties such as elasticity, stiffness, and viscosity with micrometric resolution [134]. This method involves inducing controlled tissue deformation (e.g., by mechanical or acoustic impulse or pressure change) and then detecting and analysing displacements and deformations using an OCT signal, which allows the creation of so-called elastograms—maps of tissue stiffness [135,136,137].

In the context CVDs, OCE is used, among other things, in the assessment of vascular wall biomechanics, the identification and characterisation of atherosclerotic plaques, and the examination of the heart muscle after a heart attack [132,138]. OCE allows for the differentiation of stiff tissues (e.g., calcifications, fibrous plaques) from more elastic ones, which is crucial for assessing the stability of atherosclerotic lesions and the risk of cardiac complications [134]. Experimental studies have shown that OCE enables the detection of changes in elasticity in post-infarction scars, assessment of the degree of fibrosis, and monitoring of regenerative processes in the heart [136,138].

In metabolic diseases such as diabetes or obesity, OCE can be used for early detection of changes in vascular and tissue biomechanics that precede the development of CV complications. Thanks to its high resolution and sensitivity, OCE allows for the assessment of microstructural changes in the vessel wall that are not detectable by other imaging methods [133,134,137]. This method has the potential to complement established clinical modalities in the future.

### 3.2. Biomarkers of Remodelling

In this subsection, we describe circulating ECM biomarkers, highlighting their increasing clinical utility in CV and metabolic diseases, and briefly discussing their mechanistic roles, which are primarily investigated in preclinical studies (Table 1).

Matrix metalloproteinases 2 and 9 (MMP-2, MMP-9) are enzymes from the zinc endopeptidase family that participate in the degradation and remodelling of the ECM [139,140,141]. MMP-2 (gelatinase A) and MMP-9 (gelatinase B) mainly break down type IV collagen, gelatin, elastin, and other ECM components, enabling processes such as cell migration, angiogenesis, and tissue repair, as well as tumour invasion and metastasis [142,143,144].

MMP-9 is of particularly high clinical value as a marker of tumour progression and aggressiveness, as well as a potential therapeutic target. In CVDs, especially after myocardial infarction, MMP-9 is recognised as a biomarker of cardiac remodelling and chronic inflammation [142,145]. MMP-2, on the other hand, may be associated with longevity and a favourable metabolic profile, although its role is more complex and context-dependent [146].

Under physiological conditions, MMP-2 and MMP-9 activity is tightly regulated by inhibitors (TIMPs), ensuring a balance between ECM degradation and remodelling [145]. Disturbances in this balance lead to pathological remodelling, observed in cancer, CV, and metabolic and inflammatory diseases, among others [139,142].

Elevated TIMP-1 concentrations are observed in patients with CVDs (e.g., heart failure, cardiomyopathies, hypertension, and atherosclerosis). High TIMP-1 levels are associated with an increased risk of all-cause and CV mortality, as well as with the progression of myocardial fibrosis and left ventricular diastolic dysfunction [147,148,149]. TIMP-1 is also elevated in individuals with metabolic syndrome, type 1 and 2 diabetes, and obesity, where it correlates with metabolic parameters such as BMI, waist circumference, and IR [148,150]. Studies have shown that TIMP-1 may be a sensitive and specific marker of CV risk and cardiac fibrosis, and its high concentration is a predictor of adverse cardiac events and death [149].

TIMP-2, although less frequently studied, also shows elevated concentrations in CV and metabolic diseases (e.g., heart failure, cardiomyopathies, hypertension, and metabolic syndrome) [139,148,150]. Its level correlates with organ damage, fibrosis, and metabolic parameters, as well as with arterial wall thickness and left ventricular hypertrophy [139,151]. In some studies, TIMP-2 has been found to be a predictive marker of serious CV events, although the results are less clear-cut than for TIMP-1 [152].

LOX is a key enzyme responsible for remodelling the ECM by initiating covalent cross-linking of collagen and elastin fibres. LOX and its isoforms (LOXL1-4) catalyse the oxidation of lysine residues in collagen and elastin, leading to the formation of cross-links that give tissues mechanical strength and elasticity [153,154]. Thus, LOX plays an essential role in maintaining tissue structural integrity, repair processes, wound healing, and proper organ development [155].

Dysregulation of LOX activity leads to pathological stiffening of the ECM, which promotes the development of fibrosis, CV diseases (e.g., hypertension, atherosclerosis, aneurysms, heart failure), and cancer [156,157].

In the CV system, LOX regulates vascular remodelling and influences smooth muscle cell migration, cytoskeletal reorganisation, and endothelial homeostasis. Changes in LOX expression are associated with the progression of atherosclerosis, aneurysm formation, vascular calcification, and adverse cardiac remodelling [154].

Type III procollagen, more specifically its amino-terminal propeptide (PIIINP or PRO-C3), is one of the most important biomarkers of ECM remodelling, reflecting the rate of type III collagen synthesis and turnover [158,159]. This collagen is an important component of the ECM in the heart, blood vessels, lungs, liver, and kidneys, and its excessive production leads to fibrosis and tissue stiffness [160]. Under physiological conditions, type III collagen synthesis is tightly regulated, but in chronic diseases such as heart failure, kidney disease, liver fibrosis, and lung disease, an increase in PIIINP concentration in the blood and/or urine is observed [158,159].

Elevated PIIINP levels are strongly associated with active myocardial fibrosis, progression of heart failure, adverse left ventricular remodelling after myocardial infarction, and an increased risk of death and CV complications [158,161]. In kidney and liver diseases, PIIINP correlates with the severity of fibrosis and can be used to monitor disease progression and evaluate treatment efficacy [159,160].

The determination of PIIINP in serum or urine is a non-invasive method for assessing the activity of fibrosis and ECM remodelling, and its prognostic value has been confirmed in numerous population and clinical studies [158,159,160]. Hyaluronan (hyaluronic acid, HA) is a non-sulphated glycosaminoglycan that is a key component of the ECM in many tissues, including the skin, joints, vessels, and parenchymal organs [162]. Its main functions are to maintain tissue hydration, elasticity, and integrity, and to regulate cell proliferation, migration, and differentiation processes through interactions with cell receptors such as CD44 [163,164].

Under physiological conditions, hyaluronan supports tissue homeostasis, wound healing, and protection against damage [35,52]. In pathological conditions such as chronic inflammation, liver fibrosis, pulmonary hypertension, and cancer, an increase in HA synthesis and accumulation is observed, leading to ECM remodelling and changes in its mechanical properties [165,166,167].

The concentration of HA in the blood is a recognised biomarker of active fibrosis, especially in liver diseases (e.g., cirrhosis), as well as in CVDs and cancers, where it correlates with disease progression and prognosis [52]. HA determination allows for non-invasive monitoring of ECM remodelling and pathological processes [164,165].

**Table 1:** Key ECM biomarkers, with their functions and clinical significance.

Biomarker	Function in ECM/Remodelling	Clinical Significance and Diseases	References
**MMP-2, MMP-9**	Degradation of collagen IV, V, elastin, fibronectin; promote cell migration, angiogenesis, progression of cancer and heart disease	Biomarkers of tumour progression, atherosclerosis, heart failure, diabetes complications	[36,143,145,168,169]
**TIMP-1 and TIMP-2**	Inhibition of MMP activity; maintenance of the balance between ECM degradation and reconstruction	Regulation of remodelling, biomarkers of fibrosis, heart failure, cancer, kidney disease, periodontopathy, vascular disorders	[139,169,170]
**Type III procollagen (PIIINP)**	Indicator of type III collagen synthesis, marker of fibrosis	Prediction of cardiac complications, organ fibrosis	[168,169]
**Hyaluronan**	Component of proteoglycans, affects cell elasticity and migration	Fibrosis, tumour progression, vascular remodelling	[171,172,173]
**LOX (lysyloxidase)**	Cross-linking of collagen and elastin fibres, increasing ECM stiffness	Fibrosis, tumour progression, atherosclerosis, vascular calcification, cardiac remodelling	[153,156,174,175,176]

### 3.3. Biomechanical Measurements of Blood Vessels

The measurement of arterial compliance is a key biomechanical indicator of vascular elasticity and its ability to store blood volume when pressure increases [177]. Arterial compliance reflects the relationship between volume change and pressure change in the arteries and is an important factor influencing cardiac load and blood pressure regulation [177,178]. A decrease in arterial compliance (increased stiffness) is associated with age, hypertension, diabetes, atherosclerosis, and other CVDs, and its assessment is of prognostic importance in terms of CV risk [177,179].

In clinical practice, arterial compliance can be assessed using various non-invasive methods, including pulse wave analysis, pulse wave velocity (PWV) measurement, pressure wave contour analysis, and direct measurements of changes in vessel diameter and corresponding pressure (Table 2) [177,180]. Modern techniques use tonometry, ultrasound, bioimpedance, photoplethysmography, and machine learning algorithms, among other techniques, to estimate arterial compliance based on pressure and volume signals [181,182,183]. Methods combining several data sources, such as the fusion of oscillometric and PWV measurements, are particularly promising, as they allow for a more precise and repeatable assessment of compliance [181].

Arterial compliance decreases with age and under the influence of CV risk factors, and its reduction leads to an increase in systolic pressure, cardiac load, and the risk of complications. Regular measurement of arterial compliance, especially using non-invasive methods, may be useful in monitoring disease progression, assessing treatment efficacy, and CV prevention [177,178].

PWV measurement is considered to be one of the most important biomechanical tests of blood vessels for assessing arterial stiffness and the overall condition of the vascular system. PWV determines the speed at which the pressure wave generated by the contraction of the heart travels along the artery wall [184,185]. The greater the stiffness of the vessels, the higher the PWV value, which reflects a deterioration in the elasticity of the vascular wall and is strongly associated with CV risk, the development of hypertension, complications of diabetes, and overall mortality [186,187].

PWV is most commonly measured between the carotid and femoral arteries (carotid–femoral PWV), which allows for the assessment of aortic stiffness, a key indicator of CV risk [184,185,188]. Alternative methods include measurements between the brachial artery and the ankle (baPWV), which consider both central and peripheral sections of the arteries [184,189]. The measurement involves recording pressure or flow signals at two points in the vessel and calculating the pulse wave transit time, and then dividing the known distance by this time [189,190]. Modern devices use tonometry, US, oscillometry, photoplethysmography or phonocardiography, and, increasingly, artificial intelligence algorithms to estimate PWV based on routine clinical parameters [190,191].

PWV is an indicator with high prognostic value—an increase in PWV is associated with a higher risk of CV and all-cause mortality, as well as the progression of chronic diseases such as diabetes and chronic kidney disease [186,187]. PWV measurement is non-invasive, repeatable, and increasingly widely available, but it requires standardisation of measurement techniques and interpretation of results to ensure comparability and reliability in clinical practice [184,192].

### 3.4. Summary

Modern techniques for assessing the biomechanical properties of tissues—such as US, MRE [111,113], OCE [138], and AFM [124]—combined with the analysis of ECM remodelling biomarkers [145] and arterial compliance measurements [177] form a complementary set of tools for the early, precise, and multi-level assessment of CV risk [111,113,124,138,145]. Biomechanical imaging allows the detection of subtle changes in myocardial and vascular wall stiffness that precede classic morphological abnormalities, which is important in the identification of early fibrosis, unstable atherosclerotic plaques, and diastolic changes, among other things [193]. These methods are complemented by AFM cellular analysis, which reveals mechanical changes in cardiomyocytes and ECs that are early indicators of vascular dysfunction [126]. At the same time, biomarkers of degradation and synthesis of ECM components—especially collagen and elastin—and measurements of arterial stiffness, such as PWV, reflect the systemic state of vascular remodelling and chronic inflammation, which is closely associated with the risk of CV events [145,181].

## 4. Emerging Therapeutic Perspectives

Growing evidence suggests that altered mechanical signals should not be perceived as a secondary manifestation of disease but rather as active contributors to the development and progression of metabolic and CV disease. As mechanomedicine evolves, interventions aimed at recovering mechanical homeostasis emerge, promising innovative approaches to disease management and future therapeutic strategies [194].

Pathological ECM stiffness has been widely recognised as a defining feature of fibrosis. Driven by excessive collagen cross-linking, this process has been linked to many diseases’ pathophysiology, examples being T2D, IR, MAFLD, aortic diseases, and valvular heart diseases, where progressive ECM remodelling contributes to tissue stiffening and functional impairment [13,19,27,34]. The LOX family, an enzyme group upregulated in many fibrotic tissues, catalyses collagen cross-linking, further increasing ECM stiffness. This is why LOX activity amplifies mechanotransduction signalling and sustains fibroblast-driven pathological remodelling [174,195].

Inhibition of the LOX family, especially LOX, LOXL1, and LOXL2, has been reported to suppress fibrosis and promote its reversal in rodent models of hepatic, cardiac, renal, and pulmonary fibrosis [174].

Experimental studies present therapeutic targeting of LOX, LOXL1, and LOXL2 as a promising approach to treating liver fibrosis [174]. LOXL2 is the first LOX family member to be selectively targeted, leading to the development of the monoclonal humanized antibody simtuzumab. Its effectiveness has been tested in clinical trials on patients with hepatitis C virus (HCV) and human immunodeficiency virus (HIV) coinfection, primary sclerosing cholangitis (PSC), and metabolic-associated steatohepatitis (MASH), with no clinical benefit detected [174,196]. These results may be an effect of LOXL2’s indirect and allosteric inhibition and insufficient liver scar penetration [174].

Despite the disappointing clinical outcomes of simtuzumab, other LOX family members remain promising anti-fibrotic targets. Special attention is devoted towards LOX and LOXL1, as their tissue distribution differs from that of LOXL2, and their contribution to collagen cross-linking remains significant [174].

ECM stiffness, along with cytoskeletal tension, can be modified through YAP/TAZ signalling. If dysregulated, this mechanotransducer drives fibrosis, vascular remodelling, and adipose hypertrophy—processes commonly associated with both metabolic and CV diseases [28,51].

Verteporfin (VP) is a drug that is commonly used in ophthalmological disorders and has been recently discovered to inhibit YAP. Research has shown that VP decreases nuclear YAP expression through cytosolic 14-3-3 σ, a protein binding YAP in the cytoplasm. Therefore, VP has been investigated in terms of treating multiple diseases in which YAP activity is elevated, for example, cancer, hypertension-induced kidney disease, and pulmonary fibrosis [197]. Recent studies suggest that using monocyte membrane-coated nanoparticles for targeted VP delivery to atherosclerotic plaques may decrease inflammatory gene expression and macrophage infiltration in the atherosclerotic arteries of mice (Table 3) [197,198].

Statins, a medication group widely used in patients with atherosclerosis, have been recognised to decrease YAP/TAZ activation through inhibition of its upstream activator RhoA. This results in reductions in inflammation and EC proliferation caused by disturbed blood flow. The same effect can be achieved with endothelium-restricted inhibition of NF-κB, as its activity increases in case of shear stress. Statins achieve this outcome by decreasing endothelial AKT phosphorylation [11].

As previously stated, fibrosis is a process that is widely associated with metabolic and CV diseases. There are drugs exerting anti-fibrotic properties, such as nintedanib (NTB) and pirfenidone (PFD), with mechanisms of action different than those already depicted here.

NTB is a tyrosine kinase inhibitor (TKI) shown to supress signalling pathways involved in fibroblast activation and migration, but also in excess ECM deposition—processes influenced by mechanical cues [202,203]. Clinical trials have shown that NTB decelerates fibrosis progression and improves lung function, leading to its approval for idiopathic pulmonary fibrosis (IPF) and chronic fibrosing interstitial lung disease (ILD) [202,204]. As fibrosing remodelling in different tissues has been shown to be driven by shared mechanobiological mechanisms, NTB may be proven effective in preventing CV fibrosis. As of now, preclinical trials have investigated its anti-fibrotic efficacy across different organs [202]. Moreover, research suggests that, in vascular ECs, NTB downregulates Arginase-II, which, in turn, ameliorates cellular senescence and inflammation triggered by ox-LDL. Therefore, NTB may be a potential therapeutic agent for atherosclerosis [205].

PFD is another anti-fibrotic medication approved for IPF treatment. Although its mechanism of action remains not fully understood, it is suspected to reduce the expression of pro-inflammatory cytokines (TNF-α, IL-4, IL-13) and pro-fibrotic agents (TGF-β). The suppression of TGF-β signalling indirectly affects the deposition of ECM and, in turn, tissue stiffness [204,206]. As fibrosis is one of the key factors promoting cardiac diseases’ pathophysiology, pirfenidone may become crucial in CVD treatment. Preclinical and clinical trials assessing this matter should be undertaken [206].

Understanding mechanobiological processes presents opportunities for the further development of therapeutic strategies directly modulating mechanotransduction. Tissue engineering has been shown to modulate tissue micromechanics such as stiffness and topography and, therefore, alter the behaviour of cells [207]. Combining pharmacological and biomechanical interventions holds potential for developing personalised therapies, as mechanical changes could be addressed specifically in different patients.

## 5. Discussion

The authors report a key advancement in research within the field of mechanobiology that has emerged over the past few years. The literature highlights the significant importance of mechanobiology as a scientific discipline. It plays a crucial role in an interdisciplinary context, contributing to embryogenesis as well as organismal development and function, and demonstrating strong links with regenerative medicine [208]. Recent studies emphasize the interplay of mechanical and physical factors with metabolic modifications in maintaining the proper course of cellular processes, including proliferation and regeneration [209]. The literature identifies several primary mechanical stimuli, such as ECM stiffness, extracellular fluid viscosity, hydrostatic pressure, tensile and stretching forces, and FSS [207]. Mechanical forces acting on cells—described by the authors as a key element supporting life and its formation—are also examined in the context of pathological states [5]. Examples include mechanobiology-related mechanisms described in the pathophysiology of metabolic diseases [30] and CVDs [210]. In addition, metabolic disturbances may result in tissue changes that favour the development of CVDs. Mechanobiology appears to be a potential integrative framework that could help organise processes linking these pathologies [211].

Mechanotransduction pathways have been described in relation to cardiomyocytes, EC, and adipocytes [103]. Research on adipocytes had long been limited due to the assumption that their sole function was energy storage. However, the increasing prevalence of obesity has necessitated closer examination. Observations have established that adipocytes respond to mechanical stimuli by modulating their functions, potentially contributing to disease development [28]. In obesity, excessive nutritional components promote adipocyte proliferation and hypertrophy. The resulting hypoxia and stress lead to inflammation and cytokine production, which are associated with fibrotic responses and, indirectly, with IR [212]. Adipocytes also secrete bioactive compounds that influence CV function, suggesting that abnormalities in adipose tissue may contribute to CVD risk [103]. For example, chemerin, secreted by adipocytes, acts as a chemotactic factor that contributes to inflammatory pathway activation [213].

Another study demonstrated that a distinct mechanical factor—oscillatory shear stress—can contribute to endothelial dysfunction through activation of the STING pathway [214]. Shear forces are known to elicit endothelial responses initiated by PIEZO1, subsequently mediated by Transient Receptor Potential Vanilloid 4 (TRPV4), while sustained increases in calcium levels may exert detrimental effects [66].

These are only some of the proposed mechanistic links, yet they suggest associations among mechanical interactions, inflammation, oxidative stress, and systemic consequences. Analysis of the literature enables the identification of mechanisms shared across mechanobiology, metabolic diseases, and CVDs.

Tissue engineering employs in vitro models to address these issues. In addition to two-dimensional models enabling the observation of cells exposed to precisely controlled mechanical stimuli, researchers describe 3D-printed constructs combined, for instance, with iPSC-derived cardiomyocytes. Although in vitro models are useful for studying mechanobiology, they face limitations related to system-level interactions and complexity [215]. One response has been attempts to reconstruct syncytial in vitro systems. For example, Morrissette-McAlmon et al. investigated cardiomyocyte–adipocyte interactions in coculture [216]. Even with such advanced technologies, in vitro processes cannot fully reproduce the biological complexity of living organisms. Animal models also present constraints. Murine models—cost-effective and with short reproductive cycles—display physiological differences, including in cardiac function. Porcine models, despite closer physiological resemblance, show high risk of sudden cardiac death [210]. These limitations have driven the need to develop more advanced tools capable of comprehensively studying the effects of mechanical forces.

Our analysis indicates gaps concerning the availability of models integrating mechanobiological factors with metabolism in the context of CVD. While mechanical influences on metabolism and on the heart and vasculature are extensively described independently [30,210], shared mechanistic pathways can be identified. What is needed is an integrated model demonstrating how mechanical stimuli give rise to metabolic alterations that, in turn, lead to CVD.

A potentially promising tool includes organ-on-a-chip platforms employing microfluidics, capable of reproducing mechanical tension, FSS, and other forces. Challenges remain, however, particularly in standardisation [217,218]. Proposed supplements include in silico models, although researchers emphasize barriers such as insufficient integration of cellular processes with fluid dynamics [219].

Another potential solution involves advanced artificial intelligence techniques, whose role is increasingly evident in predicting CVD progression [220]. Significant achievements include both simplified models—lumped-parameter systems defined by ordinary differential equations—and more advanced representations based on partial differential equations. This concept aligns with digital twin engineering, offering predictive capabilities and decision support [221]. The digital twin is an innovative technology lacking a single precise definition. It involves digitally replicating a patient, including their individual characteristics and specific factors. Such a duplicate serves as a simulation environment enabling the observation of disease state, treatment response, and other aspects that support more effective clinical decision-making [222]. Digital twins have the potential to revolutionize numerous medical fields, including cardiology, neurology, pulmonology, endocrinology, oncology, and surgery. In cardiology, studies already highlight their high clinical utility—for example, in drug selection, adverse event prediction, haemodynamic monitoring, and surgical planning—achieving remarkable accuracy with minimal error margins [223]. Reports in the literature describe early attempts to employ digital twin frameworks for predicting clinical outcomes [224]. Broad implementation in clinical practice, however, requires further research.

Developing a model that integrates the previously described axes of mechanics–metabolism–haemodynamics with artificial intelligence-based predictive components could open new research directions in CV risk assessment. An important element would be the combined analysis of tissue mechanical properties—measured using tools such as US and MRE, OCT, or AFM—not only alongside ECM remodelling biomarkers and arterial compliance measurements, but also with metabolic parameters such as lipid profiles, glucose levels, and adipokines.

## 6. Conclusions

1. Mechanobiology is a rapidly evolving field that elucidates the links between mechanical factors and abnormalities in metabolic and CV processes.
2. It is necessary to develop models that integrate the impact of mechanical stimuli on metabolic alterations and the resulting CV risk.
3. Enhancing such models with artificial intelligence-based predictive capabilities may initiate a new research direction in the assessment of CVD risk.

## Figures and Tables

**Table 2:** The most important non-invasive methods for assessing arterial compliance, and their clinical applications.

Measurement Method	Description and Application	References
**Pulse contour analysis**	Assessment of the shape of the arterial pressure wave; determination of compliance from the wave contour	[177,178,180]
**Pulse wave velocity (PWV)**	Indirect assessment of arterial stiffness; lower compliance = higher PWV	[177,178,180]
**Bioimpedance**	Measurement of changes in blood volume in the vessel; non-invasive and repeatable method	[182,183]
**Ultrasonography and echo tracking**	Direct measurement of changes in diameter and pressure in large arteries	[180]
**Signal fusion (e.g., oscillometry + PWV)**	Combining different data sources for greater precision and repeatability	[181]

**Table 3:** Potential therapeutic agents targeting YAP/TAZ.

Therapeutic Agent	Disease	Mechanism	References
**Verteporfin (VP)**	Pulmonary hypertension	It improves pulmonary vascular remodelling	[197,199]
	Atherosclerosis	VP inhibits macrophage infiltration due to decreased inflammatory gene expression in mice	[197,198]
	Hypertensive renal injury	It mitigates all kidney damage induced by Angiotensin II	[197,200]
	Cancer	VP decreases nuclear YAP expression through cytosolic 14-3-3 σ, a protein binding YAP in the cytoplasm	[197]
**AS-1**	Hypertrophy of the myocardium	It induces phosphorylation of LATS1, a molecule crucial for YAP signalling pathway	[197]
**XMU-MP-1**	Ascending aortic expansion and cardiac hypertrophy	It inhibits MST1, a key component of the Hippo signalling pathway	[197,201]

## Data Availability

No new data was created or analysed in this study. Data sharing is not applicable to this article.

## Abbreviations

The following abbreviations are used in this manuscript:

CV	Cardiovascular
CVD(s)	Cardiovascular Disease(s)
T2DM	Type 2 Diabetes Mellitus
IR	Insulin Resistance
MAFLD	Metabolic-Associated Fatty Liver Disease
NAFLD	Non-Alcoholic Fatty Liver Disease
PAD	Peripheral Artery Disease
CAD	Coronary Artery Disease
FA	Fatty Acids
ECM	Extracellular Matrix
IACs	Integrin Adhesion Complexes
ROCK	Rho-Associated Protein Kinase
TGF-β	Transforming Growth Factor β
GLUT1/4	Glucose Transporter Type 1/4
GSVs	GLUT4 Storage Vesicles
AKT	Protein Kinase B
FAK	Focal Adhesion Kinase
YAP	Yes-Associated Protein
TAZ	Transcriptional Coactivator with PDZ-Binding motif
TEAD	Transcriptional Enhanced Associate Domain
GSIS	Glucose-Stimulated Insulin Secretion
AMPK	AMP-Activated Protein Kinase
FGF1	Fibroblast Growth Factor 1
TRP	Transient Receptor Potential
TRPM2	Transient Receptor Potential Melastatin 2
KLF15	Krüppel-Like factor 15
EC	Endothelial Cell
FSS	Fluid Shear Stress
EndMT/EMT	Endothelial-to-Mesenchymal Transition
eNOS	Endothelial Nitric Oxide Synthase
NO	Nitric Acid
VCAM-1	Vascular Cell Adhesion Molecule-1
ICAM-1	Intracellular Cell Adhesion Molecule-1
MCP-1	Monocyte Chemotactic Protein-1
NF-κB	Nuclear Factor κB
HIF-1α	Hypoxia-Inducible factor 1α
NE	Nuclear Envelope
LOX	Lysyl Oxidase
HFpEF	Heart Failure with Preserved Ejection Fraction
VSMC	Vascular Smooth Muscle Cells
JNK Kinase	c-Jun N-Terminal Kinase
IκKβ	Ikappaβ Kinase
IRS	Insulin Receptor Substrate
α-SMA	α-Smooth Muscle Actin
ER	Endoplasmic Reticulum
AGE(s)	Advanced Glycation End Products
NOX	NADPH Oxidase
ROS	Reactive Oxygen Species
oxLDL	Oxidised Low-Density Lipoprotein
NLRP3	NLR Family Pyrin Domain Containing 3
UPR	Unfolded Protein Response
CHOP	C/EBP Homologous Protein
FFA	Free Fatty Acid
PKC	Protein Kinase C
TMAO	Trimethylamine N-oxide
SCFA	Short-Chain Fatty Acid
MRI	Magnetic Resonance Imaging
MRE	Magnetic Resonance Elastography
AFM	Atomic Force Microscopy
OCT	Optical Coherence Tomography
MMP-2/MMP-9	Matrix Metalloproteinases 2/9
PIIINP/PRO-C3	Type III Procollagen
HA	Hyaluronic Acid
PWV	Pulse Wave Velocity
HCV	Hepatitis C Virus
HIV	Human Immunodeficiency Virus
PSC	Primary Sclerosing Cholangitis
MASH	Metabolic-Associated Steatohepatitis
VP	Verteporfin
NTB	Nintedanib
TKI	Tyrosine Kinase Inhibitor
IPF	Idiopathic Pulmonary Fibrosis
ILD	Interstitial Lung Disease
PFD	Pirfenidone
TRPV4	Transient Receptor Potential Vanilloid 4
